# Assessing and comparing a DDPG model and GA optimization for a heat and power virtual power plant operating in a power purchase agreement scheme

**DOI:** 10.1016/j.heliyon.2024.e24318

**Published:** 2024-01-08

**Authors:** Ahmed Hany Elgamal, Mehdi Shahrestani, Maria Vahdati

**Affiliations:** School of the Built Environment, University of Reading, Reading, RG6 6DF, UK

**Keywords:** Machine learning, Reinforcement learning, Power purchase agreement, Solar PV, CCHP, Virtual power plant

## Abstract

This paper proposes a deep deterministic policy gradient (DDPG) model for the operation management of a solar power-based virtual power plant (VPP) having a PPA with the grid and supplying power and thermal energy to consumers. The VPP serves to balance the solar power intermittency, cover the demand whenever solar power is absent, and ensure an efficient supply of energy. The literature in this field has introduced optimization algorithms to determine the power plant's output power or heat on a rolling-horizon basis. Using the function approximation category, which involves reinforcement learning with neural networks, to solve the simultaneous thermal and power operation management of VPPs is still not well developed. The challenges imposed in this model are sourced from the non-linearity of the CCHP, the power and thermal balance constraints, and the consideration of continuous variables rather than discrete ones. A case study is simulated in Egypt to assess and compare the models. Compared to the genetic algorithm optimization, the proposed DDPG model achieved 3% more profit, 12% higher carbon dioxide (CO2) emissions, and 9% lower natural gas consumption. The DDPG solution was 57% faster than the GA. The results of the DDPG model proved that machine learning methods could outperform optimization in terms of optimality achievement and speed of solution. The DDPG improved the operation of energy storage units and was able to recognize the supply-demand operational pattern, ensuring the scalability of the VPP to cope with different energy demand levels.

## 1 Introduction

The need for renewables and clean energy deployment has been a matter of attention in the past couple of years. In the recent climate change summit in 2021, many countries promised to lower their emission levels to help limit the global temperature rise of 1.5 °C [1]. Later, in 2022, at COP27, it was obvious that no serious measures were taken, and the hope to achieve the target emissions is less optimistic [2]. It is seen in the view of this research, based on the current situation, that the abrupt transition from fossil-fuel-based power generation to 100% renewables is far from being achieved. It is also seen that pure dependency on renewables would not be the most effective solution that corresponds to highly fluctuating demand. As practical solutions, concepts such as decentralization and aggregation of different power plant technologies, including renewable sources, have attracted the focus of the recent literature. Those concepts help to mitigate the negative technical and economic effects of renewable intermittency and uncertainty [3].

The concept of microgrids, virtual power plants, energy hubs, or similar terminology used in literature, aggregating the previously mentioned DERs to manage their output proved to be efficient in achieving a common goal for these DERs [4]. This research focuses on VPPs which are grid-connected and exchange energy with the electricity grid. This goal can be set to maximize profit [5] or to minimize carbon emissions [6]. The literature related to VPPs addressed several optimization algorithms attempting to find the best fast-solving and optimal achievement of the objective function. The focus of VPPs was mostly on those operating in the “deregulated markets” where day-ahead and balancing market trading platforms are available and prices are hourly variable [4,7,8]. Arbitrage and energy trading to maximize VPPs profit is possible in these markets, where VPPs could take advantage of price differences between peak and low demand periods and schedule their output power accordingly [9].

In opposite “regulated-market” adopting countries, the economic environment to promote renewables with reliable penetration to the grid may not be well matured. In regulated markets, energy prices are fixed and predefined by the government to avoid excessively high tariffs [10]. In both regulated and deregulated markets, the government could agree to purchase renewable energy from an Independent Power Plant (IPP) at a predefined price for a certain duration under a power purchase agreement (PPA) [11]. The literature is not rich in researching VPPs operating under the PPA scheme which represents a challenging financial environment for renewables, as governments usually aim to purchase power at the lowest possible prices. For example, some countries have a good potential for solar energy but do not experience a fast growth rate due to too low electricity prices such as Egypt, Qatar, Iran, Algeria, and Vietnam holding electricity prices at 0.018 $/kWh, 0.026, 0.027, 0.038, respectively [12]. In those countries, the main challenge for the deployment of renewables is the low electricity prices and the reliance on subsidies. Exploring the constraints and the obstacles that prevent paving the road for more renewables needs to be more researched in countries adopting regulated markets.

The objective of this research is to demonstrate that machine learning could independently learn the optimal dispatch decision for simultaneous energy management of heat and power and that it could operate as optimization methods which were traditionally widely used for hourly energy management. The boundary environment of the model being investigated is a regulated market predefined energy tariff. When heat supply (i.e. thermal plant) is integrated into the energy system, a challenge arises from efficiency non-linearity, operational constraints enforcement, and the modeling of state of charge of energy storage systems. Accordingly, this research will compare the widely used dispatch optimization approach against the recently emerging deep reinforcement learning approach to find the optimal behavior of a renewable VPP covering electricity, heating and cooling demand, and aggregating energy storage systems and evaluate the outcome in terms of profit, CO2 emissions, and overall technical efficiency.

The rest of the paper is organized as follows: section 2 presents the literature review, and section 3 illustrates the problem statement and conceptual framework of the proposed VPP system. Section 4 presents the methodology, where the simulation scenarios (configurations), optimization solution method, and DDPG method are explained, section 5 presents the VPP mathematical model defining the objective functions and constraints. Section 6 presents the case study input data and demand profiles. Section 7 presents the results, the discussion, and the summary, and finally, section 8 presents the conclusion.

## 2 Literature review

The literature related to VPPs could be classified by the optimization methodology adopted to achieve the objective function, and by the aggregated power plant technologies. Consideration of simultaneous thermal energy supply from the power system, and energy storage, as a part of the optimization process, can be regarded as another parameter of the literature classification. The literature has been examined in sequential procedure; first, the papers related to aggregated power plants (VPPs or Microgrids), using linear programming optimization methods, are assessed. Second, the importance of consideration of non-linearity is assessed, and relevant papers are examined and reviewed. Third, the deployment of machine learning methods to solve aggregated power plants' energy management is examined and reviewed. The review of machine learning implementations in forecasting renewables is beyond the scope of this research. The following paragraphs will illustrate the review of relevant literature.

In many studies, conventional plants are integrated into the VPP being dispatchable and flexible in ramping up/down, accordingly, there is a requirement for a binary integer to switch ON/OFF the operation of these power plants. Considering the presence of an integer in the optimization problem, Mixed Integer Linear Programming (MILP) has been widely and traditionally used in a lot of studies but requires linearization of objective functions and constraints. Liu, Zheng [13] presented a MILP model for a VPP aggregating gas turbines, wind power, solar PVs, and flexible demands including thermal storage-powered air conditioning systems and Electric Vehicles. The model simulated uncertainties of wind by probabilistic distributions. The VPP is fed with day-ahead market price data and its management is based on 24-h electricity demand data. The study proved an increase of profit by 12% when using flexible demand. Although this study considered thermal storage in the system and that this storage is used to reflect the air conditioner power demand, the simulation of simultaneous heat and power supply from the VPP is not modeled. Zamani, Zakariazadeh [14] proposed a heat and power VPP model contributing to the real-time electricity market, aggregating a CHP plant, solar PV, wind turbines, a boiler, thermal storage, and demand response resources. MILP is used to optimize the output driven by maximizing the profit as an objective function. Point Estimate Method (PEM) was used to simulate the uncertainty of wind and loads but the penalty of deviation from the scheduled power is not modeled. The results reported the VPP profits and focused on the difference between the presence and absence of demand response resources which proved to be efficient, however, it doesn’t criticize the optimization method. Zapata, Vandewalle [15] modeled a VPP consisting of several micro-CHP and solar PVs and introduced MILP to maximize the profit from trading in the day-ahead and balancing market, considering constant efficiencies. The optimization is scheduling the VPP output according to the forecasted solar power and thermal demand, and in a real-time generation, a rolling horizon approach is adopted, meaning that the VPP reschedules the overall output to mitigate the deviation from the original day-ahead schedule and subsequent deviation financial penalties. A similar study adopting a risk aversion approach is presented by Castillo, Flicker [16] for a VPP aggregating solar PV, Battery Energy Storage Systems (BESS), solar PVs, gas engines, diesel gensets, and a fuel cell. Although MILP guarantees a single optimal solution without falling in local optima [4], it is obvious from the literature that the MILP method is not able to consider any non-linear functions such as the part-load efficiency function when considering CHP plants or dispatchable thermal plants, all non-linear functions must be linearized to either constant values or divided with piecewise approximation.

Considering non-linearity, non-linear mixed integer programming (MINLP) was used by Naval and Yusta [17], to maximize the profit of a VPP trading with the real-time electricity market. The VPP integrates a wind farm, hydroelectric power plants, solar PV plant and several pump stations having a contracted power demand (required) with the grid. Exceeding the contracted demand at specific periods negatively impacts the VPP profit function as a financial polynomial penalty function stated by the local legislative body, and this is where the non-linearity in the model originates. Ju, Zhao [18] attempted to introduce MINLP to solve a VPP model aggregating Power to Gas units, gas storage tanks, wind turbines, solar PVs, Gas Turbines, Electric Vehicles, and controllable loads, taking into consideration the quadratic function of the CGT efficiency. On the other hand, Ju, Zhao [18] declared that MINLP is complicated, time-consuming, and hard to obtain an optimal solution, and switched to MILP instead by linearizing the non-linear functions by piecewise approximation. On the other hand, Heuristic methods proved to be easier in considering non-linear objective functions or constraints and binary integers, such as Particle Swarm optimization (PSO) [19] and Genetic algorithm (GA) [20]. Maleki, Hafeznia [21] compared both GA and PSO algorithms to maximize the profit of a VPP aggregating solar PVs, a wind turbine, a fuel cell that operates for heat and power supply, and a thermal storage tank. The profit comes from the exchange with the grid. It was found that PSO is superior to GA by 0.7% only for this VPP model. However, both algorithms are stochastic and yield different results. Heuristic methods by definition do not guarantee optimal solutions and may easily fall in local optima if the solver is early terminated.

The previously reviewed methods could be categorized as model-driven approaches a VPP is managed by physical equations governing operation constraints and efficiencies. Alternatively, data-driven approaches and machine learning applications are recently being introduced in power generation-related literature. Naturally, to train the data-driven model, training data must be obtained first from the physical model. Liu, Shen [22] used Artificial Neural Networks to learn the optimal dispatch of an electric-gas coupled system consisting of gas-fired generators. The objective function was set to minimize the cost of producing gas and generating power. To provide the required training data for the model, MILP was used to generate the optimal results before feeding it to create the ANN model. The output proved much better computational efficiency for the ANN compared to model-driven optimization, however, the optimal costs for both are nearly similar.

Data-driven approaches that are based on previously trained input-output data would probably replicate the trained data and would not yield different results for the new (non-trained) data. One specific type of machine learning, reinforcement learning [23], has been recently developed in the literature to be applied in power systems, also combined with neural networks to consider continuous (non-discrete) optimization variables [24]. As will be explained later, reinforcement learning is a suitable technique that could be applied to power plant operation, since it is based on rewarding desired actions and penalizes undesired ones so that the system learns autonomously the optimal action to be taken without running an optimizer each time of its operation [25]. In this context, Liu, Liu [26] presented a Deep Deterministic Policy Gradient (DDPG) model (which is one of the reinforcement learning algorithms that relies on neural networks) combining conventional generators, wind turbines, and solar PVs. The model aims to minimize the costs of power generation coming from the deviation from forecasted power penalties and the cost of fuel. When compared to a model predictive control approach, Liu, Liu [26] found that DDPP yields lower uncertainty cost, meaning a lower deviation from the forecast. However, the method is not compared with optimization and did not include heat supply which may complicate the modeling.

Lin, Guan [27] proposed another deep reinforcement learning model for a VPP aggregating solar PVs, wind turbines, and micro-gas turbines supplying power only but did not include heat as well. Hua, Qin [28] presented a data-driven dynamic control approach based on deep reinforcement learning to optimize the energy management of a cluster of several microgrids. Compared with conventional proportional integral control and optimal power flow, the machine learning approach achieved 7.1% and 37%. Constraints of the model of Hua, Qin [28] are related to power only, the current research advances this by addressing the interconnecting heating, cooling, and power constraints and considering a CCHP with technical efficiency modeling in the problem. Other studies introduced reinforcement learning in the dispatch of power plants but mainly to improve the forecasting of uncertain parameters such as solar power, wind power, or market prices [29,30].

In terms of simultaneous heat and power, Zhou, Hu [31] presented a deep reinforcement learning model to find the optimal economic dispatch decision in the day-ahead market for an aggregated system consisting of a gas turbine acting as a CHP, boiler, wind turbine, and thermal storage system. The states of the system, meaning the scenarios that the model would encounter, are defined by feeding the wind output power, electricity demand, heat demand, and variable prices to the training agent. The objective function, which in terms of deep learning models is intended to maximize a cumulative reward, aims to minimize the operational cost of the turbine and the boiler and to minimize the purchased power from the grid (i.e. indirectly encourage selling to the grid). Due to difficulties in handling hard constraints, such as power and thermal balance, constraints are added to the reward function by assigning a high penalty for imbalance. The study however did not consider cooling demand, and overlooked detailed profit achievement definition (profit from thermal sales, selling to consumers, imbalance penalty). It also simulated only 24 h and did not model the non-linearity of the gas turbine. The details of the CHP model, such as heat-to-power ratio electric and thermal efficiencies are not illustrated. In addition, the deep learning model in comparison with the optimization method yielded a very minor difference of 0.029%, which may signify a superiority for optimization in scheduling problems. This may be also due to that prices are hourly variable and not fixed. In the same context, Gao and Lin [32] tested a deep reinforcement learning algorithm on a non-renewable aggregated system connected to the grid (which could be referred to as VPP in this case), combining an internal combustion engine (acting as a CCHP), absorption chiller, electric chiller, gas boiler, thermal storage system. The study aims to maximize the profit of energy exchange with the grid on a real-time basis and minimize the cost of CCHP operation. Compared to a rule-based control method which is based on logical judgments requiring user experience, deep learning is superior by 31% in minimizing the costs. However, the study simulated only 24 h and did not study the impact of integrating intermittent renewable power which may change the results.

As stated by Ref. [26], the research on the economic dispatch of power plants using sub-types of reinforcement learning that deal with continuous variables is limited. In addition, it is obvious that research dealing with aggregated power plants, either Microgrid or VPPs, are not directly comparable. The results depend on the type of aggregated technologies, whether connected to the grid or isolated and the type of market (regulated or deregulated) which affects the objective function driving the dispatch management.

The review of previous studies revealed considerable gaps in knowledge on evaluating and criticizing the application of reinforcement learning in the economic dispatch of VPPs in comparison with traditional model-driven optimization methods. This work seeks to advance the state of the art and address the knowledge gaps. The main contribution of the paper could be summarized as follows:

1 To the best of our knowledge, this work is the first to present a Deep Reinforcement Learning model for VPPs operating under a PPA scheme on a rolling-horizon basis and supplying both power and thermal energy. The solution provided by the RL model will be an exact optimal solution in comparison with heuristics methods which are stochastic and may fall in local optima and at the same time, are needed for consideration of model functions' non-linearity. Relevant literature did not cover the case of rolling-horizon management where pre-scheduling is not performed and the decision is done in real-time of energy supply, as well as overlooked to solve the simultaneous heat and power management which adds more constraints to the model.
2 This research develops a framework for deploying VPPs in regulated energy markets and uses the dictated government prices as a boundary condition for the model, driven by maximization of the profit, opposite to the minimum cost of energy approach which proposes a hypothetical tariff which might not be applicable sometimes unless a policy change is applied.

The above-mentioned contributions pave the way for more development in function approximation and machine learning categories of solutions that can recognize the pattern of the driving variables (e.g. power and thermal demand, energy prices). In addition, the proposed model is scalable and workable for similar patterns. The proposed reinforcement learning model is driven by the objective function tailored for renewable-energy-based VPPs working with a “Pay-as-generated” PPA scheme presented by Elgamal, Vahdati [33], which defines the relationship between the VPP and the grid in terms of power exchange. This scheme is characterized by a lower level of uncertainty, as prices are flat and known, therefore, simulating the VPP with variable market prices is beyond the scope of this paper.

## 3 Problem statement

A multi-energy heating, cooling, and power VPP is proposed to operate within a regulated market boundary with a predefined electricity tariff and energy trading PPA price. The VPP model in this research is based on balancing solar power intermittency through backing up with a dispatchable plant, storage systems, and grid power exchange. The solar power modeling is conceptualized to utilize the most available area of residential compounds to maximize the capacity of solar PV installations. From the economic aspect, this research focuses on a VPP having a PPA with the grid and the consumers, hence, acting as a mediator between the consumers and the grid where it assumes the responsibility of covering power demand and selling surplus to the grid. The output of each component of the VPP shall be driven by the objective of achieving an overall maximum profit of the aggregated system, where the revenues come from selling power and thermal energy to consumers and selling surplus power to the grid. The subtracted costs from the revenues are assumed here as the operating costs of the dispatchable plant (i.e. fuel consumption).

Non-renewable backup power generation plants and energy storage are required to be deployed as more solar or wind power becomes integrated into the grid, otherwise, their intermittency may cause blackouts. Sørensen [34] suggests that for less than 100% renewable energy, conventional power plants can be used for backup of intermittency, but for 100% renewable targets, energy storage would be necessary. However, due to their high capital cost, battery storage implementation is limited. Conventional plants on the other side are not emission-free, In the best scenario, natural gas-fired engines would be considered clean energy suppliers as their CO2 emissions are lower compared to other fuels. Waste heat from the combustion process of gas engines, if not utilized efficiently, is emitted into the environment representing a wasted resource of energy. Therefore, the combined heat and power (CHP) plants concept is introduced, to utilize waste heat for the process of domestic space heating, cooling, hot water, cold water, and industrial heat requirement coverage. The plant could be either engine-based (internal combustion engine, Stirling engine, gas turbine) [35] or fuel-cell [36].

Based on the previous explanation, the VPP model in this research aggregates a natural gas-fired CCHP, double effect direct-fired absorption chiller, thermal storage systems (heating and cooling), rooftop solar PV panels, and Battery Storage System (BSS). Solar power is assumed to be rooftop units occupying approximately 60% of the buildings' rooftops [37]. As CHP is used in the model, normally the space heating and cooling will be assumed to be covered by the waste heat from the CHP, which reflects on the modeling by constraining the sum of output of the CHP plant and thermal storage units to be higher than the thermal demand. That means that there may be non-utilized waste heat evaded into the atmosphere. On the other hand, the sum of generated power from CHP, solar PVs, dispatched power from BESSs, and power purchased from the grid should be equal to the sum of power demand and the surplus power sold to the grid.

**Fig. 1:**
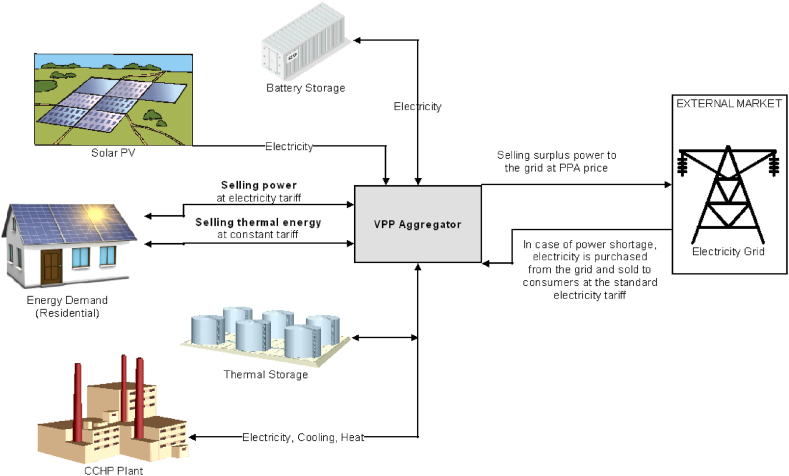
VPP model conceptual diagram [33].

Therefore, the model assumptions and conceptual framework be summarized as follows as illustrated in Fig. 1:

- The VPP operates solar PVs, CCHP, BESS and TES systems
- The VPP sells surplus power to the grid at the PPA price of solar power
- The VPP sells power to residential consumers at the electricity tariff price,
- Thermal energy, covering space heating and cooling, is supplied to consumers at an assumed price equal to the solar power PPA price
- Solar PVs area are assumed to occupy all potential available buildings rooftop areas
- If the VPP is not able to cover the power demand, it acts only as a mediator to purchase and supply electricity from the grid with no overhead profit, consumers pay the same electricity tariff but to the VPP manager and the VPP does not make a profit. The VPP makes a profit only from selling its own generated power or thermal energy to consumers, and from selling the surplus power to the grid at PPA price.

## 4 Methodology

### 4.1 Baseline case-genetic algorithm

**Fig. 2:**
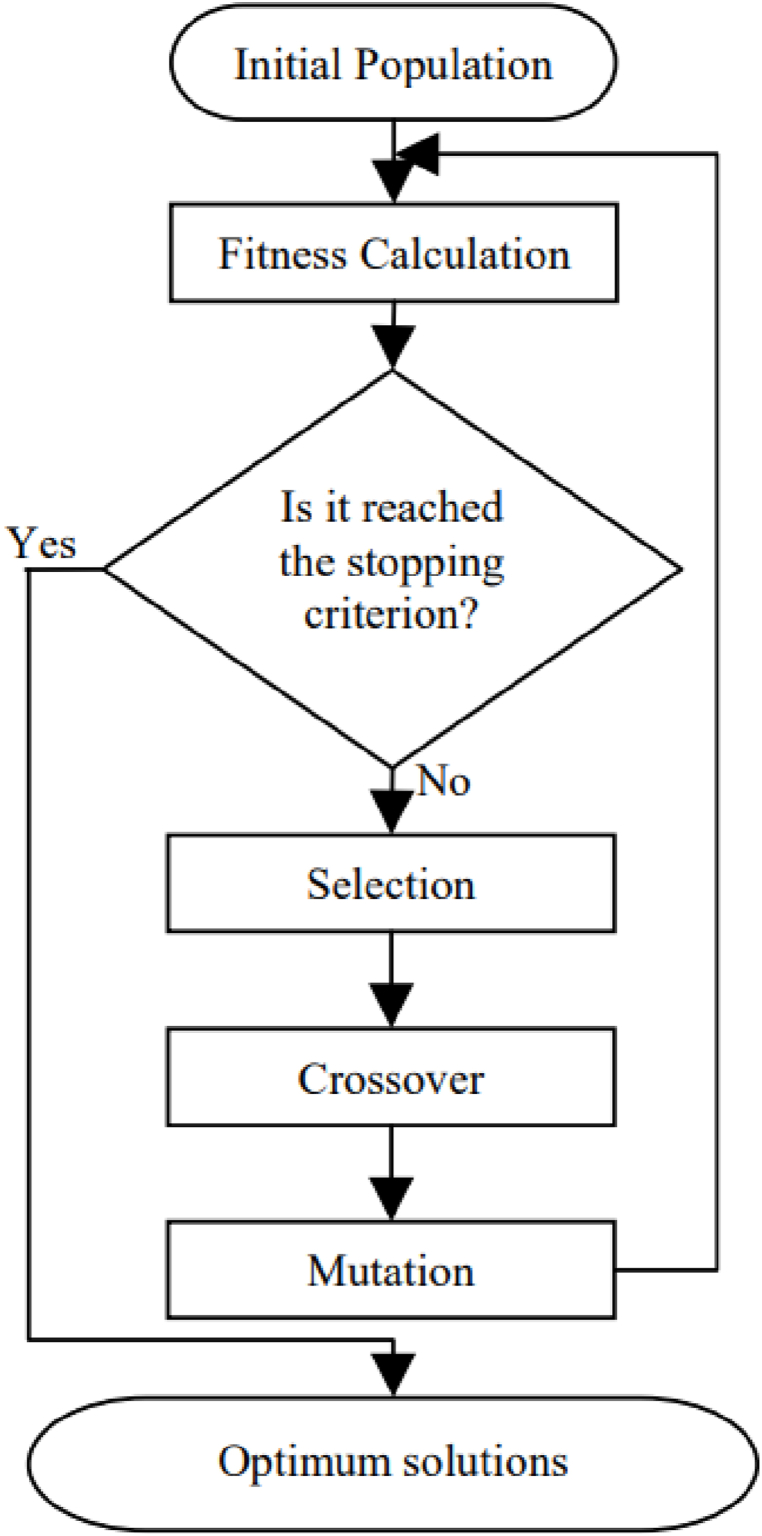
GA general framework [38].

Genetic Algorithm belongs to the heuristic algorithms category which can provide an optimal solution to problems having integers or non-linear constraints or objective functions. A general framework for GA is shown in Fig. 2. It is, however, falling easily in local optima with difficulties of knowing whether or not it is yielding an optimal solution. As explained before, conventional power plants (such as gas engines used in this model), will require binary integers to represent the ON/OFF of the system. In addition, CCHP part-load efficiency is non-linear, therefore, GA is used as a baseline to be compared to the other reinforcement learning method which will be explained in the next section.

### 4.2 DDPG

Power plants driven by demand follow a certain pattern of operation, basically depending on the electricity and the thermal demand. If market prices are known or predefined, as in our PPA case, the pattern driving the power plants remains similar but differs in scale from winter to summer. In that case, it is deemed suitable in this research to find a method to recognize the different scenarios of operation leading to the formation of the pattern driving the plants and building its own rule-based operation rather than performing optimization each time step. However, when market prices are hourly variable and differ from day to day, this driving pattern is not the same every day so it will be computationally expensive to recognize it. After the plant derives the rules of operation leading to optimal results, it should work under a similar pattern. This is considered a part of data-driven modeling, as the system output is programmed according to the fed-in data and not according to the physical operational model.

**Fig. 3:**
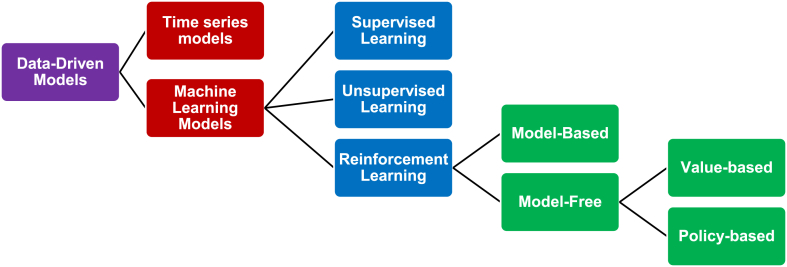
Data-driven models categories [39,40].

Data-driven modeling is a powerful approach to solving nonlinear and complex data structures (models having complicated relationships between input and output) (Gupta and Singh, 2021). It relies on historical data for training a model to extract useful information and correlate input and output data, then the model becomes knowledgeable enough for decision-making (predict the output) (Gupta and Singh, 2021). As shown in Fig. 3, data-driven models could be classified as time-series models (mainly based on statistical regression), and machine-learning models. Time-series models are usually useful in forecasting a single variable, accordingly, they are not so efficient in hybrid systems with multi-variables. Machine learning (ML) models are a fast-developing field and proved to be useful in different applications.

Reinforcement learning (RL) does not require external training data such as historical input-output data, which would make this approach in favor of the dispatch optimization problem. It builds its knowledge by an iterative approach which is similar to how the human gains his experience, which means that the model generates its training data and trains itself [41]. This is achieved by using a system of reward and penalty for the model when it performs correctly or incorrectly, respectively. If the learning subject (environment or agent) performs a certain action deemed as constructive, it gets rewarded for doing this action or for reaching its ultimate goal (delayed reward), and if it performs a negative action it gets penalized, in this way, the environment builds its knowledge through adopting the constructive or positive actions and avoiding the non-constructive ones [42]. As a general concept of RL, the learning subject (namely “agent”) interacts with the “environment” and selects a certain “Policy” (decision or strategy) to perform an “Action” to move from a “State” to another state (transition), where the ultimate goal is to maximize the cumulative sum of “Rewards” arising from these actions [43]. To select the optimal policy that returns rewards rather than penalties, the agent may go through many iterations and experience penalties and rewards. From trial and error, the agent learns which optimal policy to follow to achieve the maximum sum of cumulative rewards [44]. The fact that this approach is sequential (moving from one state to another) and that the model learns from iterations without the need for prior data makes this method the most suitable for this research among the other machine learning techniques.

As shown in Fig. 3, RL agents could be categorized as model-based and model-free [40]. Q-learning is the most popular value-based agent resembling a V-lookup table that aims to fill in an action value for each parameter corresponding to the state of the environment. The policy-based category aims to find the policy that achieves the optimum value of the objective function. In the context of this research and for a better understanding, the action for example could be the value of the generated power from the plant. The state is a combination of the parameters that affect the action, for example, the combination of power demand, thermal demand, and price at a certain time step. It is obvious from this explanation that the action and state spaces are too large to be considered discrete variables or a lookup table to be filled in. Accordingly, deep neural networks are coupled with RL agents to face the challenges arising from a large set of possible combinations or non-linearities of environment relations.

**Fig. 4:**
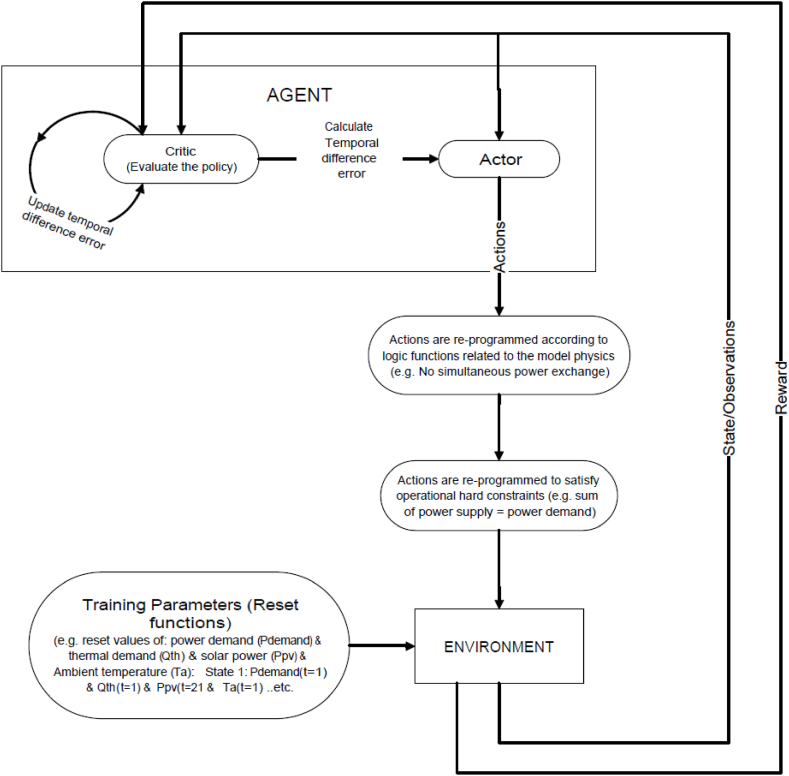
Proposed DDPG framework.

Deep Deterministic policy gradient, employed in this research, is a policy-based RL agent, it uses an “actor-critic” neural network for function approximation to learn a Q-function and a policy [45]. As shown in Fig. 4, the principle of actor-critic is where the actor produces actions values and feeds them to the environment to predict the next state, then the environment returns the predicted values, calculates the reward, and feeds it back to the critic which evaluates the predicted values and calculates the error [46]. A reset function is fed to the environment to define variables that represent scenarios that the environment encounters. During training, at any time step (t), the reset function assumes random values from 0 to the maximum value of each governing variable (power demand, thermal demand, state of charge of BESS and TES) of the training dataset.

**Fig. 5:**
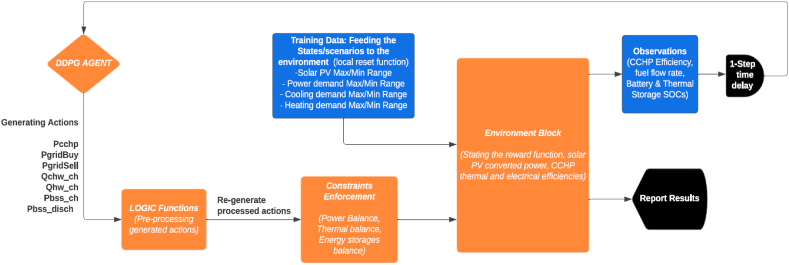
DDPG model application conceptual framework.

The model is implemented with Matlab R2022a. The agent and the environment are modeled in Simulink. A conceptual framework for the Simulink model is shown in Fig. 5. The process goes from left to right where initial actions (decision variables) are assumed by the agent, then they are introduced to the constraint enforcement block where the upper and lower constraints along with hard constraints (e.g. sum of power supply equals the demand) must be satisfied. Next, the variables go to the environment block which mainly states the reward function which is defined in this paper as the profit function, however, from many iterations, the reward appears to be calculated without overshoots when it is minimized or at best, normalized, therefore, the profit function is divided by the power demand to reduce the calculated reward values. As the RL still has a limitation of introducing integer variables which are needed to prevent simultaneous contradicting actions, such as charging the BESS when it is discharging, a “LOGIC” block is placed between the RL Agent and the constraints block. This “LOGIC” block defines conditional if-then functions, stating that if the discharging power variable is higher than zero the charging power variable equals zero, the same for TES charging and discharging variables. In addition, a condition is written that when the power is purchased from the grid, the power sold to the grid is enforced to zero. The next section illustrates the objective functions and constraints in detail.

## 5 Mathematical model

The modeling formulas and demand data are taken from the model prepared by Elgamal, Vahdati [33], where this work is a further expansion of the method used to reach a better solution. The objective function and model constraints are explained in the next paragraphs.

The objective function aims to maximize the profit that comes from the following:

- Selling power to consumers at standard electricity tariff which they are used to pay normally. This is equivalent to the electricity consumption (dead) at $\lambda_{el,t}$ price; however, the shortage in power supply purchased from the grid and directly sold to consumers is subtracted from the profit: $\left(P_{demand,t}-P_{gridbuy,t}\;.\;u_{grid,t}\right)\;.\;\lambda_{el,t}$.

The VPP in this case is only mediating between the consumer and the grid without receiving profits.

- Selling surplus power, mostly solar power, to the grid on a “pay-as-generated” PPA basis: $\left(P_{gridsell,t}\;.\;u_{gridsell,t}\right)\;.\;\lambda_{PPA,t}$.

It is assumed here that any generated surplus power is sold to the grid at PPA price. The PPA price is defined in the model as the value set by the government for solar PV project bidders.

- Selling thermal energy to consumers equivalent to their cooling and heating demand, at the same PPA price of solar PV: $\left(Q_{CHW\_demand,t\;}+Q_{HW\_demand,t\;}\right)\;.\;\lambda_{th,t}$.

The thermal tariff is assumed in this paper because the concept of selling district energy has not yet been implemented in many countries.

- The costs subtracted from the revenues equal to the cost of natural gas used to fire the CCHP: ${\dot{m}}_{f,t}.C_{f}$.

The model is subjected to operational constraints and upper/lower bounds for the variables representing the equipment capacities. The energy balance constraints are stated such that the sum of the power supply.

The decision variables of the simulation are the CCHP output power, BSS charging/discharging power, TES charging/discharging energy, and power purchased/sold from/to the grid. Binary integers are added to control the ON/OFF statuses of CCHP, thermal storage, BSS, and Sold/Purchased energy to/from the grid. The binaries are only used in the GA model as decision variables, however, in the RL model, those binaries are replaced with logic conditions (if-then) (e.g. if $P_{gridbuy,t}$ >0 ; then $P_{gridsell,t}=0$)

The optimization objective function (which is the same as the GA fitness function) is stated as shown below in Equation (1). The same equation is also used as the reward function of the DDPG model:

(1) $$\sum_{t=1}^{T}Profit=\sum_{t=1}^{T}\left(P_{demand,t}-P_{gridbuy,t}\;.\;u_{grid,t}\right)\;.\;\lambda_{el,t}+\left(P_{gridsell,t}\;.\;\left({1-u}_{grid,t}\right)\right)\;.\;\lambda_{PPA,t}+\left(Q_{CHW\_demand,t\;}+Q_{HW\_demand,t\;}\right)\;.\;\lambda_{th,t}-\left({\dot{m}}_{f,t}.C_{f}\right))$$

Subjected to (constraints):

(2) $${SU}_{t}=v_{t}\left(1-v_{t-1}\right);\;t=1,\ldots ,T$$

(3) $$v_{t}\in \left\{0,1\right\};\;t=1,\ldots ,T$$

(4) $$u_{grid}\in \left\{0,1\right\};\;t=1,\ldots ,T$$

(5) $$u_{cchp}\;x\;P_{CCHP_{\min}}\leq P_{CCHP,t}\leq {u_{cchp}\;x\;P}_{CCHP_{\max}};\;t=1,\ldots ,T$$

(6) $$0\;<\;P_{gridbuy,t}<\;P_{gridbuy\_max}$$

(7) $$0\;<\;P_{gridsell,t}<\;P_{gridsell\_max}$$

The state of Charge for TES and BSS is estimated as follows [47]:

(8) $$E_{CHW-TS,t}=\left(1-\varepsilon_{t}\right).E_{CHW-TS,t-1}+u_{CHW-ch}\;{.\;Q}_{CHW-TS-ch,t}-{u_{CHW-ch}\;.\;Q}_{CHW-TS-disch,t}\leq E_{CHW-TS\_max,t}\;;\;t=1,\ldots ,T$$

(9) $$E_{HW-TS,t}=\left(1-\varepsilon_{t}\right).E_{HW-TS,t-1}+u_{HW-ch}\;.\;Q_{HW-TS-ch,t}-u_{HW-disch}\;.\;Q_{HW-TS-disch,t}\leq E_{HW-TS\_max,t};\;t=1,\ldots ,T$$

(10) $$E_{BSS,t}=E_{BSS,t-1}+u_{BSS-ch}\eta_{charge}P_{charge,t-1}-\;u_{BSS-disch}\left(\frac{1}{\eta_{discharge}}\right)P_{disch,t-1}\leq E_{BSS\_max,t};\;t=1,\ldots ,T$$

(11) $$u_{CHW-ch}+u_{CHW-disch}\leq 1\;;\;u_{CHW-ch},u_{CHW-disch}\;\epsilon \;\left\{0,1\right\}$$

(12) $$u_{HW-ch}+u_{HW-disch}\leq 1\;;\;u_{HW-ch},u_{HW-disch}\;\epsilon \;\left\{0,1\right\}$$

(13) $$u_{BSS-ch,t}+u_{BSS-disch,t}\leq 1\;;\;u_{HW-ch},u_{HW-disch}\;\epsilon \;\left\{0,1\right\}$$

Output from solar power and PV panel efficiency are estimated as follow [48]:

(14) $$P_{PV,t}=\eta_{PV}A_{PV}G_{t};\;t=1,\ldots ,T$$

(15) $$T_{c,t}=T_{o,t}+\left[\left(\frac{NOCT-20}{800}\right)\right]\;.\;G_{t};\;t=1,\ldots ,T$$

(16) $$\eta_{PV}=\eta_{r}\;x\;\left[1-\beta \left(T_{c,t}-T_{ref}\right)\right];\;t=1,\ldots ,T$$

Power and thermal demand balance constraints are estimated as follows:

(17) $$u_{grid,t}.P_{gridbuy,t}+P_{CCHP,t}+P_{PV,t}+u_{BSS-disch,t}.P_{disch,t}-u_{BSS-ch,t}.P_{ch,t}-\left(1-u_{grid,t}\right).\;P_{gridsell,t}=P_{demand,t};\;t=1,\ldots ,T$$

(18) $${COP}_{c}.\left({\gamma \;.\;P}_{CCHP,t}+{\dot{m}}_{f-ac,t}\;.\;{LHV}_{f}\right)+\eta_{TES}\;.u_{CHW-disch}\;.\;Q_{CHW-TS-discharge,t}-\eta_{TES}\;.\;u_{CHW-ch}\;.\;Q_{CHW-TS-charge,t}\geq Q_{CHW-demand,t}\;;\;t=1,\ldots ,T$$

(19) $${COP}_{h}\;\left(\gamma \;P_{CCHP,t}\right)+\eta_{TES}\;.u_{HW-disch}.Q_{HW-TS-discharge,t}-\eta_{TES}.u_{HW-ch}.Q_{HW-TS-charge,t}\geq Q_{HW-demand,t};\;t=1,\ldots ,T$$

(20) $$Q_{recovered}=Q_{exh}+Q_{jw}=\gamma \;.\;Pcchp$$

Part-load electrical efficiency modeling of the CCHP is expressed as a function of nominal efficiency, estimated as follows [49]:

(21) $$\eta_{CCHPe\_nominal}=0.0194\;\ln\left(P_{CCHP_{\max}}\right)+0.2321$$

(22) $$\eta_{CCHPe,t}=\eta_{CCHPe\_nominal}\;(0.1024\;{\left(\frac{P_{CCHP,t}}{P_{CCHP_{\max}}}\right)}^{3}-0.7332\;{\left(\frac{P_{CCHP,t}}{P_{CCHP_{\max}}}\right)}^{2}+1.0155\;\left(\frac{P_{CCHP,t}}{P_{CCHP_{\max}}}\right)+0.6153$$

(23) $${\dot{m}}_{f,t}=\frac{P_{CCHP,t}}{\eta_{CCHPe,t}.\;LHVf}\;;\;t=1,\ldots ,T$$

The CO2 emissions are calculated as per the following formula [50]:

(24) $$F_{CO2,total}=30\;x\;\left(\sum_{t=1}^{T}{\dot{m}}_{f,t}.{LHV\;}_{f}.\mu_{CO2_{NG}}+\sum_{t=1}^{T}{\dot{m}}_{f-ac,t}\;.{LHV\;}_{f}.\mu_{CO2_{NG}}+\sum_{t=1}^{T}P_{gridbuy,t}\;.\;\mu_{CO2_{grid}}\right)$$

Equations (2), (3), (4) state the binary integers for the start-up and shut-off of the plant. Equations (5), (6), (7) defines the lower and upper limits of the CCHP plant, the power to be purchased from the grid and the power to be sold to the grid. Equations (8), (9), (10) depict the TES and BSS charging and discharging power in relation to the state of charge. Equations (11), (12), (13) defines binary integers for the TES and BSS to avoid simultaneous charging and discharging. Equations (14), (15), (16) defines the solar power conversion from irradiation, area, and panel efficiency (equation (16)) which is a function of the cell temperature (equation (15)). Equations (17), (18), (19) describes the power and thermal balances. It should be noted that power balance is formulated as an equality constraint, however, thermal balance is formulated as inequality, which means that the thermal energy supply must be always equal to or higher than the thermal demand. Equation (20) defines the heat-to-power ratio which is assumed in this research as 1. Equations (21), (22)) define the nominal and instantaneous electric efficiency of the CCHP. Equation (23) defines the mass flow rate of the natural gas input for the CCHP, which is a function of the efficiency.

CO2 emission from the CCHP and the power purchased from the grid are calculated as per equation (24) using the below-shown inputs [51] :

• CO2 emission factor from the grid $\mu_{CO2\_grid}$ = 0.923 kg/kWh
• CO2 emission factor from natural gas $\mu_{CO2\_NG}$ = 0.220 kg/kWh

The next section presents the case study and the result of the simulation.

## 6 Case study

**Fig. 6:**
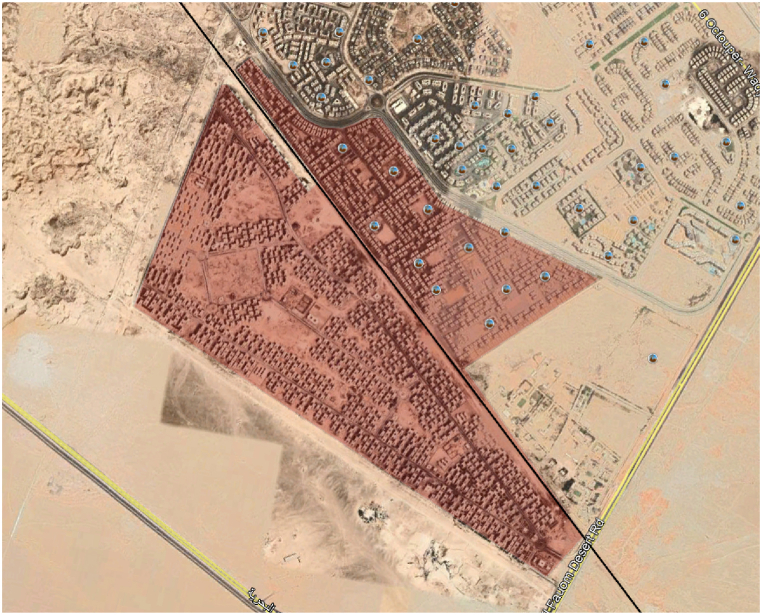
Case Study boundaries.

**Fig. 7:**
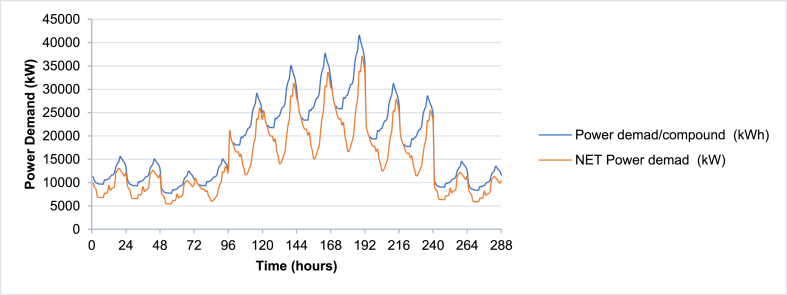
Gross and net Power demand profile. Net Power demand excludes the cooling and heating load.

**Fig. 8:**
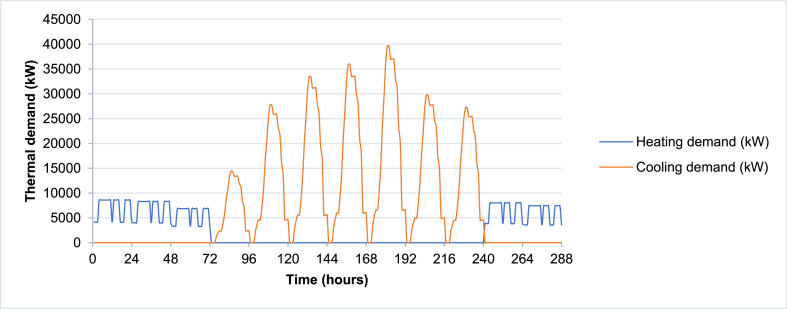
Space cooling and heating demand.

The VPP simulation model is tested using the case study location used in Elgamal, Vahdati [33] (as shown in Fig. 6) and the power and thermal demand are used as inputs to the model accordingly as shown in Fig. 7, Fig. 8 respectively. Table 1 illustrates the basic inputs for the model. The case study site consists of 1185 blocks, each having 24 apartments and approximately 304 m^2^ flat roof. In this case study, it was assumed that 60% of the total roof area is used for PV installation, accordingly, the solar PV area is 216,144 m^2^. CCHP capacities are assumed to have an upper limit of 30,000 kW and the models will calculate the actual maximum output of the simulation period. TES and BSS unit capacities (kWh) are initially assumed to be 3 times the maximum CCHP size, with charging and discharging upper limit equal to the CCHP size, meaning that the storage systems could substitute the CCHP for covering the power or thermal demand for 3 h.

**Table 1:** **–** Input parameters.

Parameter	Value
Electricity tariff	1.45 EGP/kWh
Grid exchange PPA price	0.61 EGP/kWh
Thermal tariff	0.61 EGP/kWh
Absorption chiller COP	1.4
TES & BSS roundtrip efficiency	90%

Electricity tariffs in Egypt are normally divided by ranges of consumptions, in this research, a fixed tariff is taken as an input which is the maximum value in the public tariff slices. The model is simulated on hourly resolution for 12 days that corresponds to each month, accordingly, the simulation period is 288 time steps. The model is prepared and simulated with MATLAB R2022a, on an MSI computer with an Intel® Core™ i7-9750H 2.6 GHz processor.

## 7 Results

### 7.1 Genetic algorithm – baseline model

**Fig. 9:**
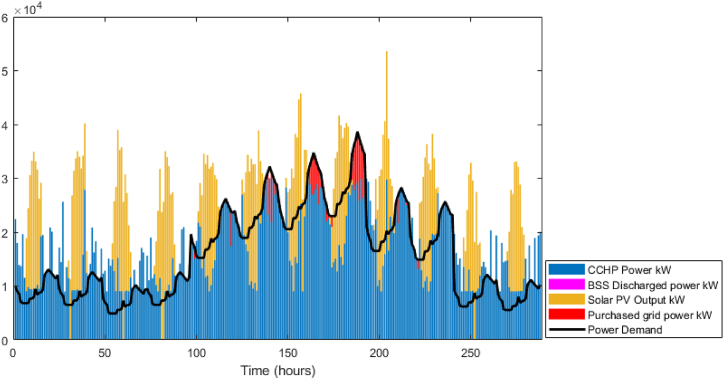
GA results -Power balance.

**Fig. 10:**
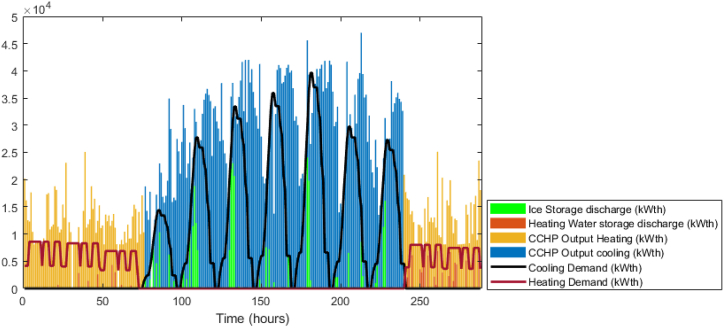
GA results -Thermal balance.

**Fig. 11:**
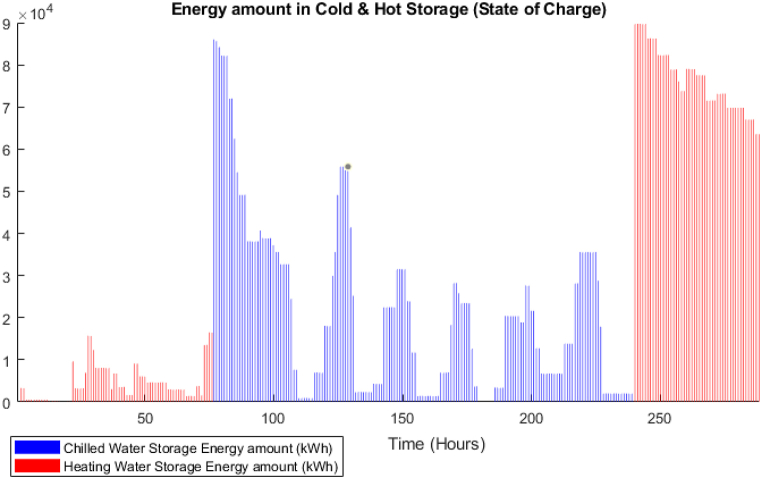
GA results -TES State of charge.

**Fig. 12:**
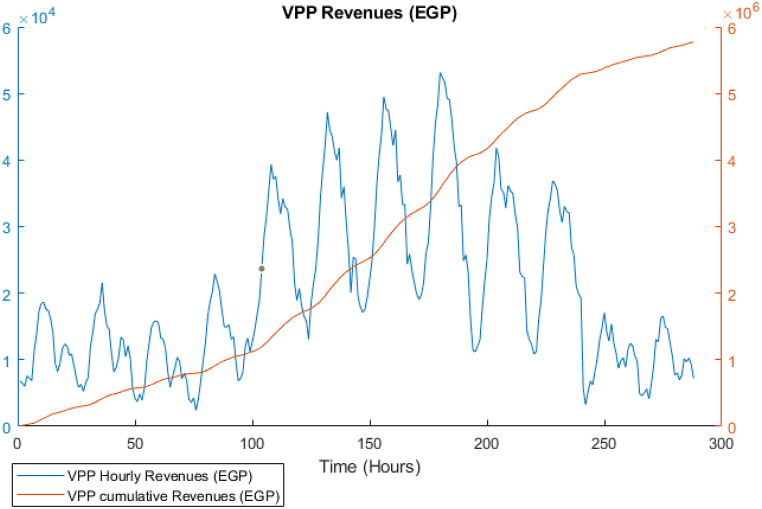
GA results - Instant and cumulative profit.

The GA optimization is performed to establish the baseline case. The power balance is shown in Fig. 9, in that figure, it is obvious that the solution preferred to utilize the CCHP power during most of the periods including peak times. However, between time steps 150–200, there is power purchased from the grid as the maximum capacity of the CCHP is defined as 30 MW. The BSS was not used at all, the system sold all the available surplus directly to the grid. From the thermal balance shown in Fig. 10, the CCHP waste heat was used to cover the thermal demand but due to that the system preferred to enforce the CCHP to follow the power demand, there were many periods where the waste heat exceeded the thermal demand, which represents a portion of the heat that is dumped to the atmosphere (i.e. whatever thermal output exceeding the thermal demand is dumped to atmosphere). The TES state of charge is shown in Fig. 11, the heat energy storage is minor and it is mostly discharging during the winter time, but operates in a charging/discharging cycle in the summer period where it is charging during times of low demand and discharging during peak times. The achieved profit for these 12 days is 5.77 Million EGP, which will be compared to the RL model. The total simulation time was 391 s in the fastest case. The profit results are shown in Fig. 12, where as expected, the profit is steadily increasing and nearly following the power supply output pattern. The major drawback of the GA model is the stochasticity of the results and the variation of each run from the other.

### 7.2 DDPG model

**Fig. 13:**
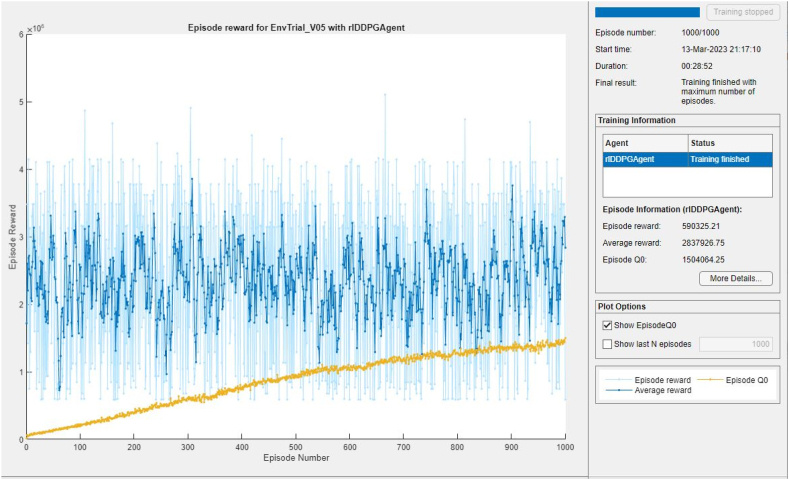
**–** DDPG Training episodes reward.

In this scenario, the DDPG model was fed 48 h (representing 1 day in summer and 1 day in winter) as training data. As shown in Fig. 13, the training was done for 1000 episodes to estimate the policy function. An episode consists of a combination of random values of power demand, thermal demand, and different random values of solar power, representing situations that the model could encounter and from which the policy function is built.

From the policy being established, the model can schedule itself when it encounters any new state of the environment, on condition that a similar pattern of input data occurs. Episode Q0, representing the value function (critic), is steadily increasing and approaching the average reward which signifies that the agent’s learning of the policy is being improved.

**Fig. 14:**
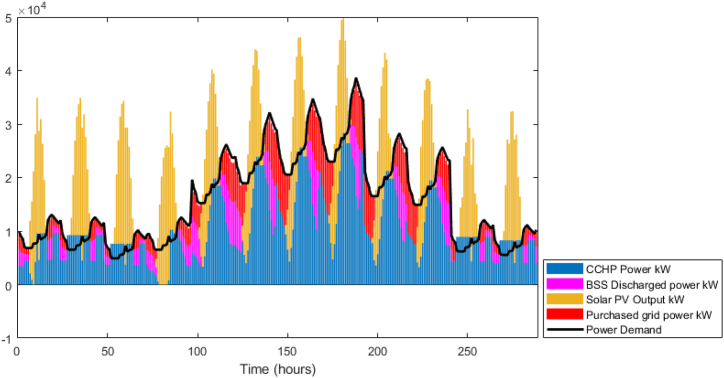
DDPG model results - Power balance.

Opposite to the GA model, as shown in the power balance in Fig. 14, the model prefers to minimize the operation of the CCHP to reduce the fuel cost, and at peak periods it prefers to purchase the shortage from the grid. Also, despite of assigning a maximum capacity of the CCHP as 30 MW, the simulation reported a maximum CCHP output of 28.3 MW, which signifies that the system sizing is performed efficiently compared to the GA model. Following the power balance, the thermal balance, as shown in.

**Fig. 15:**
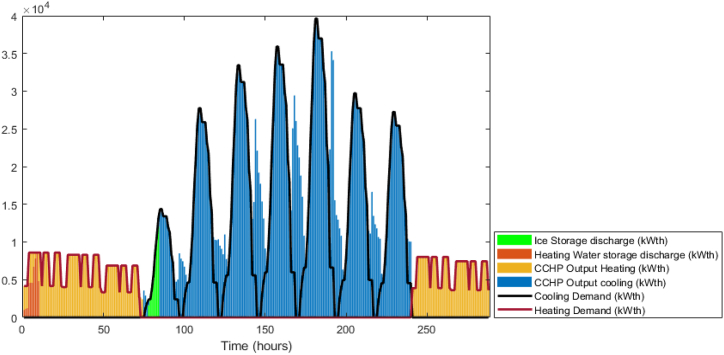
DDPG model results - Thermal balance.

**Fig. 16:**
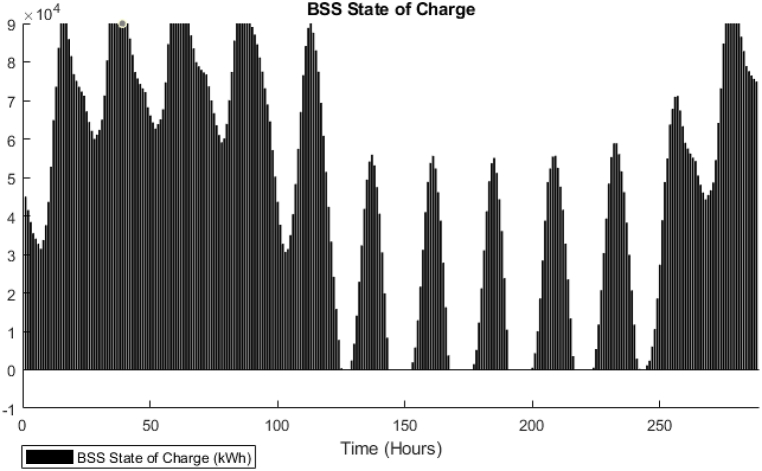
DDPG model results - BSS State of charge.

**Fig. 17:**
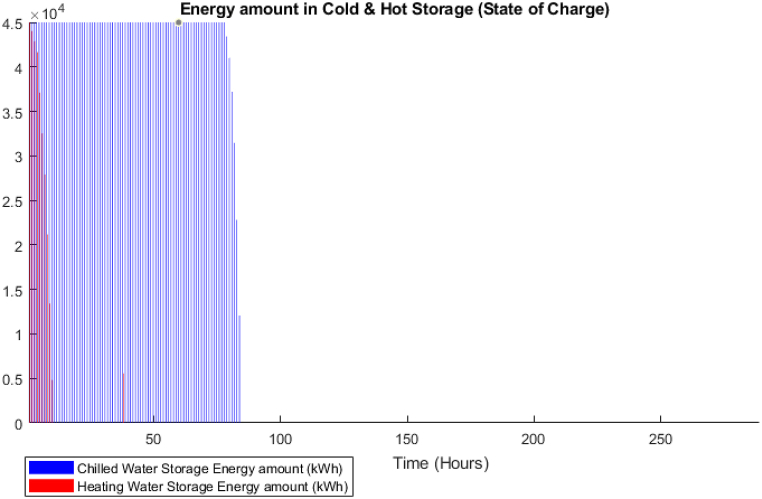
DDPG model results - TES State of charge.

Fig. 15 shows that the waste heat from the CCHP is attempting to follow the thermal demand curve which signifies that the model is attempting to minimize the CCHP output to reduce the fuel cost. In addition, the BSS output is active in this model, Fig. 16 shows the state of charge of the BSS which depicts that it is going through charging and discharging cycles at low demand/peak periods respectively. However, obviously, for the TES case, it was not the same as BSS, as shown in Fig. 17, the model is minimally dependent on the discharged thermal energy from the TES. Both heating and cooling water storages are discharged when the CCHP power is not needed, this occurs whenever the power demand is low and the solar power is available.

**Fig. 18:**
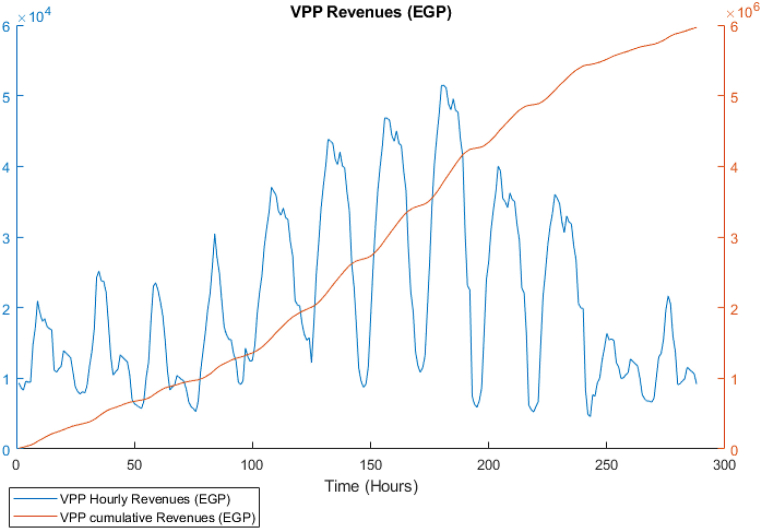
DDPG model results - Instant and cumulative profits.

Fig. 18 shows the instantaneous and cumulative profit for the RL model, which is nearly similar to the GA result but higher in the cumulative value. The achieved total profits for the 12 days is 5.9 Million EGP, which is higher than the GA model by approximately 3%. Nevertheless, the dependency on the grid is approximately 6 times compared to the GA model, since the reward in the RL model is solved cumulatively and the policy function naturally is inferred from attempting to achieve maximum cumulative profit and not instantaneous reward, which is the opposite to the case of the GA that solves the model for each time step separately. It can be also noticed that, not only that the RL model outperform the GA model in the results, but the speed of simulation is 170 Seconds, which is faster than the GA by 57%. Another advantage of this model is that it is scalable for the same demand pattern, which means that it could be applicable regardless of the location of the case study. However, in case the market prices are variable and not fixed like the PPA price in this paper, it will be required to re-run the training and redefine the prices as an additional variable in the reset function to contribute to the combination of variables that represent states of the model environment.

### 7.3 Comparison & discussion

Table 2 illustrates the overall results for the entire year, as the simulation was made for 12 days representing 1 day for each month of the year, the results are multiplied by 30 to enable the comparison between the GA and RL models. In terms of profit, the RL model is performing better than GA by 3%, however, in terms of emissions, the RL model is causing 12% more emissions than the GA due to higher dependency on the grid. This case is occurring because of the price challenge in this case study, where the electricity prices are relatively low compared to the generation costs of the CCHP. However, the CO2 emission was not in the scope of the reward or targeted to be minimized. As the input value for the CO2 emission of the grid is 0.932 g compared to 0.222 g of the natural gas, the emissions of the RL model would be lower if the grid mostly depended on gas-fired power plants rather than high emissive plants. If this were the case, it would make sense to depend on the grid for purchasing shortage of power at peak times, to help reduce the operational load on the CCHP, as well as avoid producing more waste heat that exceeds the thermal demand which would be a dumped useful energy resource. The fuel consumption is about 9% lower in the RL model since a lower CCHP output is achieved. Eventually, a higher solar fraction was achieved in the RL compared to the GA model.

**Table 2:** Annual energy produced from each unit, CO2 emissions, and profit.

SCENARIOS	Total Energy from CCHP (MWh)	Total Energy from Solar PV (MW)	Total Energy from BSS (MWh)	Total Energy sold to the Grid (MWh)	Total Energy purchased from the Grid (MWh	Annual CCHP CO2 Emission (Tons)	Annual Grid CO2 Emission (Tons)	Total CO2 Emissions (Tons)	CCHP gas consumption (m3)	Profit (EGP)
GA MODEL	153,824	64,607	0	84,663	4135	80.6	3.8	84,4	1,098,964	173,3 Million
RL MODEL	88,257	64,607	16,915	37,065	22,931	74.1	21.1	95,2	1,010,894	178,9 Million

The difference in the energy storage operation in the GA and the DDPG models is notable. In the GA, it was clear, as shown in Fig. 11, that the TES units are operating on a daily cycle and contributing to partial coverage of the thermal demand, however, the BSS is not operating. On the other hand, in the DDPG model, as shown in Fig. 16, the BSS is operating on a daily cycle, while as shown in Fig. 17, the TES units are discharging at the beginning of the occurrence of the relevant thermal demand and do not recharge. The strategies adopted in each model are different, obviously, the GA avoided utilizing the grid power, and therefore, the CCHP operation secured enough waste heat surplus to charge the TES units. The DDPG model focused on maximizing the reward which caused the CCHP operation at many time-steps to reduce its output and import more power from the grid, which was more profitable than depending on the CCHP. This strategy of the DDPG did not secure enough surplus waste heat for charging the TES units, while the surplus power was enough for the cyclic charging/discharging of the BSS.

In relation to the literature, similar studies have been compared to assess the results of this research. Lin, Chang [52] studied the energy management of a Microgrid combining solar PV, Batteries, and wind turbines, assessed with DDPG and compared with PSO and with an experience-based energy management system (a rule-based approach based on a user experience). In their best cases, the PSO and DDPG achieved 1941.28 $ and 1898.49 $ which proves that DDPG achieved better results by 2.2% higher than the PSO. Lin, Chang [52] did not consider thermal plants or covering thermal energy demand. In line with Lin, Chang [52], the presented model in this research is superior to the GA by 3% with considering thermal energy balance constraints, which advances the state-of-the-art. Additionally, compared to the case studies on the Egyptian market, many researchers approached the solution of microgrids or energy hubs by estimating hypothetical COE values to be applied on the market, such as Diab, Sultan [53] achieving 0.217 $/kWh [54], achieving 0.218 $/kWh, El-Sattar, Sultan [55] achieving 0.33 $kWh, while the current tariff 1.45 EGP/kWh (0.04 $/kWh). The proposed approach and framework of energy exchange in this research achieved a high profit using the existing electricity tariff rather than proposing a different value.

## 8 Conclusion

The energy management of multiple aggregated assets controlled by a VPP has been extremely important for achieving optimal profits. The definition of the economic framework in which a VPP is operating, whether it enables it to trade energy in the free market or predefines its relation with the grid, is crucial to properly manage the output of each asset. This paper proposed and analyzed an alternative approach to dispatch optimization, commonly used in most of the VPPs-related papers, which is meant to manage VPP output in deregulated markets for trading energy in day-ahead or real-time platforms. This paper proposed and analyzed an innovative deep reinforcement learning approach for modeling a VPP that is selling energy to consumers and the surplus to the grid at a fixed-price PPA. The new approach is different than traditional optimization methods since it is based on the function approximation concept. This new approach is deemed suitable as the governing parameters driving the output of the VPP are following a certain operational pattern with constant prices. As a result, energy management rules are learned from trial and error and reinforced by a reward and penalty system, which is the basic concept of reinforcement learning.

The new model is trained on the energy demand and solar power values, being inputs to the model, and used the training to learn a policy function to drive the model under any input data. The proposed model of this research achieved a better economic result compared to GA, faster solving time, but less efficient result in terms of CO2 emissions. However, as argued in the result section, minimizing the CO2 emissions was not the intention of the model. A reward function could be designed to consider performance or emissions objectives. The RL model encountered some limitations that could be developed further. First, the training requires a high number of episodes, and it takes 28 min to be trained on 48 points of the data. This requires a high computational efficiency to speed up the training process. Second, applying constraints, especially hard constraints, needs to be developed to consider mixed integers and continuous variables. In this research, integers were substituted by conditional statements for the relevant variables, before being fed to the constraint block.

This research calls for further work to better understand the behavior of the RL model under different situations, this shall include the following:

- Considering a different statement for the thermal balance constraint where a thermal comfort parameter could be added as a metric for excess waste heat or shortage of thermal demand coverage, instead of always keeping the thermal energy supply higher than the demand.
- Training and simulating the model under hourly variable pricing such as in balancing or real-time deregulated market prices
- Targeting the energy efficiency of the CCHP as an objective and analyzing the reward function accordingly
- Comparing the RL model with another baseline model of a different optimization category (rather than heuristic algorithms), such as MILP

## Data availability statement

Data will be made available on request.

## CRediT authorship contribution statement

**Ahmed Hany Elgamal:** Writing - review & editing, Writing - original draft, Visualization, Software, Methodology, Investigation, Conceptualization. **Mehdi Shahrestani:** Validation, Supervision. **Maria Vahdati:** Validation, Supervision.

## Declaration of competing interest

The authors declare that they have no known competing financial interests or personal relationships that could have appeared to influence the work reported in this paper.
